# ‘Don’t Worry, Honey: It’s Cooked’: Addressing Food Risk during Pregnancy on Facebook Italian Posts

**DOI:** 10.3390/foods10102484

**Published:** 2021-10-17

**Authors:** Valentina Rizzoli, Giulia Mascarello, Anna Pinto, Stefania Crovato, Mirko Ruzza, Barbara Tiozzo, Licia Ravarotto

**Affiliations:** 1 Istituto Zooprofilattico Sperimentale delle Venezie, Actually at Department of Communication and Social Research, Sapienza University of Rome, 00198 Rome, Italy; 2 Istituto Zooprofilattico Sperimentale delle Venezie, 35020 Legnaro, Italy; apinto@izsvenezie.it (A.P.); scrovato@izsvenezie.it (S.C.); mruzza@izsvenezie.it (M.R.); btiozzo@izsvenezie.it (B.T.); lravarotto@izsvenezie.it (L.R.)

**Keywords:** food risk communication, food behavior, social network, social sharing of emotions, text mining

## Abstract

During pregnancy, women exposed to microbiological risks are more susceptible to contracting specific pathogens, which can lead to serious diseases both in the mother and the foetus. Food-borne diseases can be avoided to a large extent by following good practices of food manipulation and cooking. Safe eating behaviours are influenced by knowledge and perception of food risks and are constructed, among others, online. Pregnant women often use Web 2.0 to obtain and share pregnancy-related information as a strategy of collective coping with emotions through conversations. This paper explores how knowledge and perceptions of food risks during pregnancy are shared among users on Italian Facebook pages and groups. The corpus, including 648,399 items (i.e., posts), was analysed: (a) first, by means of the Reinert method, to verify to what extent issues concerning food risks are debated; and (b) second, through a manual content analysis, to observe how food risks are addressed in terms of contents and social sharing of emotions. The main results show that food risk is not among the most discussed topics, and the least known and debated food risks are the most widespread (e.g., campylobacteriosis). Sometimes, food risks, when addressed, were minimised or denied, and the belief to be ‘less at risk’ than peers for such risk (i.e., optimistic bias) was observed. The results underline the importance, for health institutions, of building a tailored communication strategy on food risks during pregnancy to promote correct food behaviours by exploiting social networks.

## 1. Introduction

### 1.1. Food Risks during Pregnancy

Pregnant women are one of the groups most at risk of contracting food-borne diseases [1]. Due to changes in the immune system during pregnancy women are more exposed to microbiological risks in general, and more susceptible to contracting specific pathogens such as Toxoplasma gondii, Listeria monocytogenes, and Salmonella, which can cause serious diseases in women and their foetuses [2]. Previous Italian studies [3,4] recommended actively screening for toxoplasmosis during pregnancy, due to the high incidence of this infection. The results of these studies suggest that most of the pregnant women living in the areas with considerable infection rates did not develop antibodies to toxoplasmosis. Epidemiological data on salmonella describe a steady spread from 2012 to 2016 (n = 4138 in 2016, with a notification rate of about 7 × 100,000/cases per year). The incidence of listeria in 2016 demonstrated an increasing trend, though in Italy, only 3% of contagions involved pregnant women [5]. Nevertheless, the incidence can be underestimated due to the non-systematic application of the diagnostic investigation of events associated with pregnancy [5].

Food-borne diseases can be avoided to a large extent by following good food handling and eating practices (i.e., food behaviour) [6,7]. For example, listeriosis can be avoided by eliminating foods such as raw meat, raw milk, and dairy products derived from it; toxoplasmosis can also be prevented by thoroughly washing raw vegetables, avoiding raw meats or sausages, and cross-contamination. Knowing the beliefs and practices adopted by pregnant women is fundamental to rectifying possible incorrect behaviours. A study conducted in northern Italy showed both high awareness and a lack of knowledge on food safety [8]. Even though the majority of pregnant women (95.2% of the interviewed sample) supported the importance of food safety, more than half of them felt they were not sufficiently informed. Other studies have highlighted the fact that some of the most common food risks are not well known. For example, pregnant women know little or nothing about toxoplasmosis [9] and listeria [10]. Knowledge and perception of food risks during pregnancy contributed to delineate attitudes and behaviours towards them; therefore, pregnant women represent an essential turning point for study [11,12].

### 1.2. Constructing Online Knowledge and Perception of Food Risk

Faced with a myriad of information on food risk, the consumer must interpret complex and sometimes uncertain or conflicting information as a coherent message [13]. This construction of meaning takes place in everyday communication that happens in a twofold step: from institutions, journals, experts, etc. (top-down), and from laypeople [14]. This information is often extracted through social networks, a particularly accessible place of exchange. Web 2.0 has transformed how people communicate and collect information: users can both gather or create content simply and autonomously and share them. The Internet has become the preferred channel for finding information on food risks and for sharing food practices [15,16,17,18]. Pregnant women also use Web 2.0 to obtain and share pregnancy-related information without the supervision of health care professionals [19], thereby increasing the risk of spreading misinformation [20]. Even though they consider healthcare workers a reliable information source, they use social networks to seek information on food risk [10].

In recent years, attention towards the use of social media in food safety issues has increased. Social media plays a pivotal role in the impact of risk communication on food safety. As shown by Wu [21], it is possible to identify the socio-psychological factors that are key determinants for risk communication, such as risk perception, emotions, and social trust. Similarly, social media offer the possibility to observe the cognitive, affective, and behavioural responses to food safety crises. Mou and Lin [22], for example, examined the use of a microblog platform and showed how its consultation was related to the general and factual awareness of food safety incidents and preventive actions. Moreover, the authors found that the emotional response towards food safety incidents predicted both food safety risk perception and prevention action, underlining the role of social media in shaping knowledge, perceptions, and consequently behaviours.

### 1.3. Social Sharing of Emotions on Social Media

People not only search for information online, but also share it with others [23]. Due to a real or perceived risk, people face salient emotions with which they need to cope; social sharing of emotions [24,25] is a strategy of collective coping through conversations. This allows managing emotions, since, as explained by Rimé et al. [25] p. 147, “when beliefs are challenged, the basic feeling of security is undermined, and people are likely to search for social support and coping assistance. By sharing emotions with significant members of their social environment, people can find external support for their emotional work, facilitating and strengthening their coping attempts.”

Social networks serve the function of sharing emotions [14] because they reach a broad and targeted audience around a particular emotional experience [26]. Moreover, they represent the perfect environment to study the phenomenon of social sharing of emotions; through online sharing, socially apprehensive users can bypass the obstacles of direct communication [27]. This is particularly true in the case of pregnant women, who suffer a high level of social expectation with regard to their parenting/pregnancy knowledge: on social networks, they can communicate with others in the same condition and share doubts about topics they are supposed to know the answers to (i.e., food behaviours during pregnancy).

Various studies have focused on food risks during pregnancy, but only a few targeted pregnant women’s awareness and knowledge of these risks [28] and how these risks are shared on social media in the Italian context.

To summarise, it has been argued that pregnancy is a period that deserves special attention to food risk issues and social networks constitute an essential ground for building knowledge and sharing emotions, and these aspects are linked to behaviours and risk perception. Exploring these aspects can provide relevant suggestions for the construction of effective communication and intervention strategies related to food risks targeted to pregnant women.

### 1.4. Aim

The overall aim of this study is to explore how and to what extent knowledge and perceptions of food risks during pregnancy are shared on social networks (Facebook in particular). Two specific research questions lead the study:

1. To what extent is food risks during pregnancy addressed on Facebook?
2. How is it addressed in terms of content and social sharing of emotions?

## 2. Materials and Methods

The present study is part of computational social sciences and adopts a mixed research design combining quantitative and qualitative analyses of textual data (i.e., social media posts). First, we performed a quantitative analysis of the collected texts, to automatically individuate the topics (i.e., topic detection) and select those of interest (i.e., food risks related). Second, with a qualitative analysis, we identified the psycho-social processes emerging from the exchanges contained in the posts.

**Figure 1:**
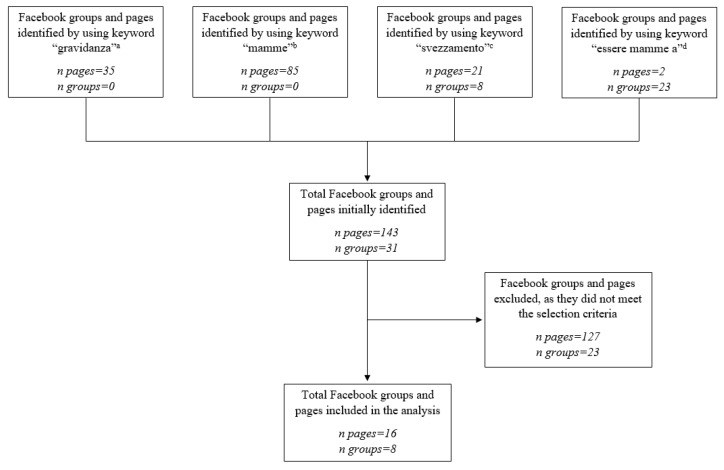
Diagram of groups/pages selection process (^a^ “Pregnancy”; ^b^ “Mothers”; ^c^ “Weaning”; ^d^ “Be mothers in”).

Facebook groups and pages related to pregnancy were identified and selected for the study. Groups and pages were selected by the authors between 18 September 2017 and 21 September 2017 by searching the following keywords on Facebook’s search option: “Pregnancy”, “Mothers”, “Weaning”, “Being mothers in…”. As shown in Figure 1, 143 pages and 31 groups were initially individuated. Only pages and groups operating in Italian (first selection criterion), publicly available to anyone with a Facebook account (second selection criterion), with at least 300 members/likes (third selection criterion), and with at least one published post in the last three months starting from the date of the selection (fourth selection criterion), were considered in the study. A total of 16 pages and eight groups were found for the study.

The 24 Facebook groups and pages finally selected are listed in Table 1.

All the contents (posts and comments to posts) published in the selected Facebook pages or groups from 28 September 2017 to 27 February 2018 were gathered. The data collection was carried out through the WebLive application (http://www.web-live.it, (accessed on 5 March 2018); cf. [29,30]), a cloud-based web platform developed by Extreme Srl.

A total of 648,399 items were collected. The corpus (i.e., the total amount of posts and comments) includes 15,857,869 occurrences (N) and 178,918 distinct forms (V). The type/token ratio (the number of distinct forms divided by the total number of occurrences) is 1.13, and the number of hapaxes (words that appear only once) stood for 52.58%. The corpus had a satisfying redundancy [31,32,33] (which is fundamental in an approach based on word count) with a type/token ratio less than 20%, even though the hapaxes exceeded 50%; this was due to the type of texts, which include grammatical errors.

Through TaLTaC2 software [34,35], the corpus was normalised by replacing uppercase letters with lowercase. Then, after a manual check, the most informative multi-words (meaningful sequences of words)—according to the relative I.S. index [34]—with frequencies more than 20, were individuated.

### 2.1. Individuating Food-Related Risks during Pregnancy SUB-Corpus

To answer the first research question—to what extent food risk during pregnancy is addressed—we identified the different topics covered in the corpus and especially those concerning food-related risks. The Reinert method [36] implemented in the IRaMuTeQ software [37] was performed (i.e., the first quantitative analysis of the data). The topics were defined by Reinert [38] as “lexical worlds”, which are clusters of words that refer to a common meaning, identified based on their co-occurrence in portions of texts (in this case, corresponding to the entire post or comment).

**Figure 2:**
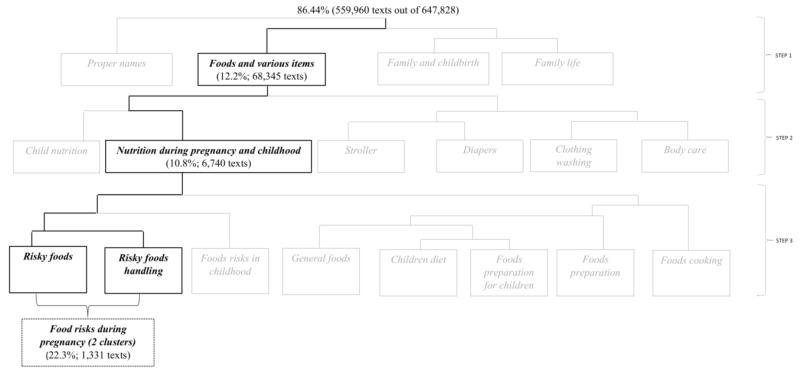
Steps for the selection of the food risks related corpus.

Once the macro-clusters were identified, we proceeded in successive steps to restrict the field of content to a sub-corpus that mainly contained the topics of interest. It was achieved by repeating the analysis in the subcorpora formed by inherent topics (cf. Figure 2). We observed the words in the clusters in their context (the portions of texts) to better understand the topic and label each cluster.

### 2.2. Exploring the Contents on Food-Related Risks during Pregnancy

To answer the second research question of how food-related risks during pregnancy are addressed in terms of contents and social sharing of emotions, both automatic and classical (manual) content analyses [32] were performed (i.e., the second quantitative and qualitative analysis of the data). First, by means of the Reinert method applied to the topics individuated in the sub-corpus, four classes were automatically identified. Then, through Atlas.ti software (version 6.6.1, Scientific Software Development GmbH 1998–2020, Berlin, Germany), two researchers involved in the project team and qualified in social research and data mining independently and manually analysed the posts/comments associated (according to the chi-square index) to the classes identified through automatic analysis (for a total of 728 posts). Only three classes out of four among the ones selected through the Reinert method were included in the qualitative analysis, since they had enough statistically significant words associated (according to chi-square index; *p* < 0.0001), and covered topics relevant to the research interests. The researchers carefully read each post/comment (a) identifying the contents which dealt with food risk and those which omitted the topic and coding argumentations related to risk perception (i.e., in which terms the risk is mentioned), cf. Elo and Kyngäs [39]; (b) analysing the texts in terms of social sharing of emotions (cf. Sarrica et al. [14]). The researchers regularly consulted each other, cross-checking the proposed codes and discussing the analyses and the interpretations, until an agreement was reached.

## 3. Results

### 3.1. The Addressed Food-Related Risks during Pregnancy

The Reinert method that was applied to the entire corpus identified four classes (or topics), which together represented 86.44% of the posts/comments (559,960 out of 647,828). As shown in Figure 2, only the class “foods and various items” was selected and analysed through the Reinert method (first procedure step). The sub-corpus (12.2% of the classified posts/comments) was composed of 68,345 posts/comments and showed good redundancy: N corresponded to 1,318,266, V to 39,073, the type/token ratio accounted for 2.96%, and hapaxes stood for 50.49%. The Reinert method applied to such corpus individuated six topics, among which the one labelled “nutrition during pregnancy and childhood” (10.8% of the previous sub-corpus; 6740 posts/comments) was selected and analysed (second procedure step; cf. Figure 2); redundancy results were adequate (N = 144,112; V = 10,261; type/token ratio = 7.12%; hapaxes = 52.09%). Eight topics were identified from the sub-corpus (second step) and subdivided by hierarchical descending analysis in two macro groups: one containing three topics related to food risks (labelled “Risky foods”, “Risky food handling”, and “Foods risks in childhood”), the other consisting of five topics related to nutrition in general. From the three topics related to food risks, the one pertaining to food risks in childhood was excluded from the analysis. The remaining two topics were selected (third procedure step; cf. Figure 2) as sub-corpus (together 22.3% of the previous sub-corpus; 1331 posts/comments) and labelled ‘Food risks during pregnancy’ after reading the most associated words to such topics, which allowed to define the content. The lexicometric measures showed that the redundancy of the final sub-corpus remained adequate, even though the number of hapaxes exceeded 50% (Table 2). The sub-corpus “Food risks during pregnancy” corresponds to 0.2% of the entire corpus (1331 posts out of 648,399).

### 3.2. Contents on Food-Related Risks during Pregnancy

**Figure 3:**
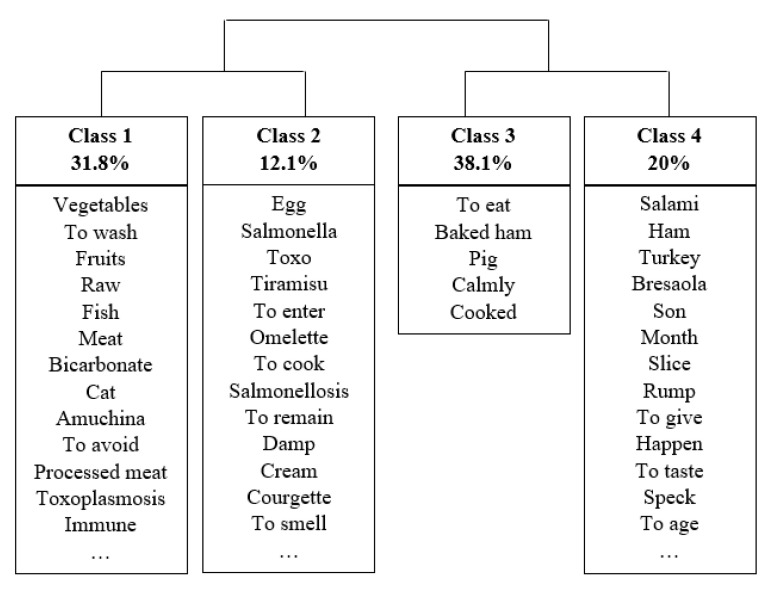
Dendrogram of the classes related to food risks during pregnancy. The most associated words listed are ordered by the chi-square index.

To better explore the content, we first performed an automatic analysis. From the “Food risks during pregnancy” sub-corpus, the Reinert method individuated four classes/topics (Figure 3) that correspond to 86.85% of the posts/comments (1156 out of 1331).

Topic 1 (31.8% of the classified posts/comments) included words referring to toxoplasmosis, potential transmission, and how to avoid it (e.g., *toxoplasmosi/toxoplasmosis, verdura/vegetables, carne/meat, crudo/raw, gatto/cat, amuchina, lavare/to wash*). Topic 2 (12.1% of the classified posts/comments), similar to the previous one, contained words referring to salmonellosis and foods that can transmit it (e.g., *salmonella, uova/eggs, frittata/omelette, tiramisu*). Topic 3 (36.1% of the posts/comments) included only a few words significantly associated—the most representative one was mangiare/to eat—referring to the permitted practices. Topic 4 (26.5% of the posts/comments) included words referring to cold cuts (e.g., *salame/salami, prosciutto/ ham, mortadella/bologna, bresaola/air-cured beef, and prosciutto cotto/ham steak*).

To search for how the risk is addressed and social sharing of emotions, a manual analysis of the texts was performed. Only three out of four classes were manually analysed in the study, since one (topic 3) had only a few words associated with it and not enough meaningful content (it only contained words and segments related to the act of eating in general). Through this analysis, the researchers identified whether the content had been correctly conveyed or not (label “confusion or misinformation”), the presence of references to experts, and two psychological processes: optimistic bias and social sharing of emotion. Each label is illustrated below.

#### 3.2.1. Confusion or Misinformation

The three macro-categories manually analysed (topics 1, 2, and 4), included references to food (*cold cuts*) and mainly two infectious diseases: salmonellosis and toxoplasmosis. Other infections (e.g., listeriosis and campylobacteriosis) were only marginally treated mainly in the context of the discourses related to the two above-mentioned infections.

Even though posts in topic 4 mainly refer to cold cuts’ consumption during pregnancy, some posts (less-strongly associated according to the chi-square index) concerned children’s cold cuts consumption. Most of the posts reported that it is not possible to eat raw cold cuts (mainly due to the risk of contracting toxoplasmosis). However, certain aspects that emerged from some posts reflected confusing knowledge and required investigation. In some posts, the portion size of raw cold cuts was related to the greater or lesser possibility of contracting an infectious disease. In these posts, it was claimed that “by eating a limited amount of raw cold cuts nothing happens”.

*Per 2 fette di crudo non capita nulla il problema era se ti fossi mangiata tutta la coscia di crudo/For two slices of raw [ham] nothing will happen to you; the problem would have existed if you had eaten the whole leg [of ham].*

*Post associated with topic 4*

Doubts emerged about the ageing of the raw cold cut.

*Per chi non lo sapesse il prosciutto crudo viene stagionato per circa 13 mesi ed è proprio per questo che non è considerato un problema/For those of you who do not know, raw ham is aged for about 13 months, which is why it is not considered a problem.*

*Post associated with topic 4*

Regarding salmonellosis-related topics, most posts correctly state that it is possible to contract it by eating raw eggs; however, some controversial issues and knowledge gaps emerged. A confusion among salmonellosis and toxoplasmosis in terms of how it is possible to contract them was detected. This result indicated a lack of knowledge about the infections.

*Ma scusate con le uova crude io sapevo che puoi prendere la toxo da quando in qua non è più così?/Wait, I thought raw eggs gave you toxo, since when is this not true anymore?*

*Post associated with topic 2*

Moreover, the practice of washing the eggshell to avoid contamination from salmonella was suggested.

*[…] Se ci si preoccupa per la salmonella è presente nel guscio quindi si lava bene con spugna, acqua e sapone […]/[…] If you are worried about salmonella, it is present in the eggshell, so wash it well with sponge, water, and soap […]*

*Post associated with topic 2*

In the topic related to toxoplasmosis, the associated posts mainly referred to how it is contracted. They also generally mention the consumption of raw foods, including fish. Although some posts report accurate information, misinformation regarding how toxoplasmosis is contracted was detected.

*Ma con gli affettati non c’entra niente la toxo, ma la listeriosi, sono due cose diverse e indipendenti tutti possono prendere la listeriosi, per le donne incinte molto pericolosa, se si è immuni alla toxo puoi mangiare le verdure crude senza problemi ma comunque non puoi mangiare carni crude e formaggi a latte non pastorizzato/But toxo has nothing to do with raw cuts, but listeriosis, they are two different and independent things everyone can take listeriosis, very dangerous for pregnant women, if you are immune to toxo you can eat raw vegetables without problems but you cannot eat raw meats anyway and non-pasteurised milk and cheeses.*

*Post associated with topic 1*

#### 3.2.2. The Reference to the Expert

The arguments sustained to approach food risks during pregnancy were analysed and classified. The expert figure is sometimes mentioned in support of the argument. This figure is most often the gynaecologist.

*[regarding toxoplasmosis] La mia ginecologa ha detto che c’è più rischio con verdure e frutta non lavate bene, quindi coccola il tuo micetto/my gynaecologist said there is more risk with unwashed vegetables and fruit, so pet your kitten*

*[regarding toxoplasmosis ] La carne sempre ben cotta, verdura e frutta ben lavate, tutto ciò sotto consiglio del mio ginecologo, quindi stai tranquilla: se sono cotti in forno puoi mangiare tutto stasera. Buona serata/Meat has to always be well cooked, and vegetables and fruit well washed, all of these indications are the advice of my gynaecologist, so don’t worry: if they are cooked in the oven you can eat them all tonight. Good evening*

#### 3.2.3. Optimistic Bias

Another argument that was often mentioned was the rhetorical ‘I’ve always done it, nothing ever happened’. The knowledge of the presence of certain risks is displayed; however, this is minimised by the experience of the author of the post.

*Io ho mangiato tutto, pure il pesce crudo, e ho tre bimbi perfetti/I have eaten anything, even raw fish, and I have three perfect children.*

These types of arguments can be attributed to a process known as optimistic bias (i.e., the belief to be less at risk than their peers for negative events; [40]). Individuals tend to think that a lack of issues in the past may ensure less exposure to the same risk in the future [41].

*Ma la torta di mele puoi mangiarla è cotta, è il tiramisù con le uova crude che non si può mangiare. Comunque io due gravidanze mangiato di tutto i miei figli sanissimi/But you can eat apple pie—it is cooked, it is tiramisu with raw eggs that you cannot eat. Anyway, I have been pregnant twice and I ate everything; my children are very healthy.*

#### 3.2.4. Social Sharing of Emotions

By observing all the above-illustrated arguments and the topics addressed, it is possible to notice that, in general, the posts are mainly (a) expressions of doubt and concerns, and (b) answers to such doubts and concerns, as seen from the text below.

*Tesoro stai tranquilla anche se fosse stato prosciutto crudo le ha messe in cottura friggendole e quindi non c‘è più il problema, la toxo si prende solo con affettati crudi mangiati così, ma se li cuoci non danno problemi/honey, don’t worry, even if it were raw ham, it was fried and therefore there is no problem anymore, the toxo is contracted only with raw meats eaten as they are, but if you cook them, they don’t give problems*

These expressions mirror the process of social sharing of emotions with a specific interpersonal dynamic [42]. An episode that elicited a particular reaction (that is a concern in most of these cases) is shared in a peer group. A person who perceives similarity (e.g., “I am pregnant too”; “Dear friend, I am in the same condition”) feels connected and is willing to help, providing support by answering (e.g., “Don’t worry, there is no problem anymore”). This triggers a process of emotional recovery. Another example of this process is provided below.

*Le primissime settimane non c’è passaggio con embrione per cui stai tranquilla la natura è perfetta. Ora che lo sai invece stai attenta perchè basta una fetta di salame o di prosciutto o una verdura cruda non lavata/ In the very first weeks [of pregnancy] there is no passage with the embryo, so don’t worry, nature is perfect. Now that you know, however, be careful because a slice of salami or ham or an unwashed raw vegetable is enough.*

## 4. Discussion

Social networks have a pivotal function in building knowledge and perceptions of people. In this paper, we investigated to what extent, and with respect to what contents, knowledge of food risks during pregnancy is derived from the exchanges on Facebook.

After a selection of the contents gathered, we observed that food risks are addressed in the social network, but in a small proportion. Moreover, even when food risks are discussed, some microbiological risks are more frequently treated than others (particularly salmonellosis and toxoplasmosis rather than listeriosis), while others like campylobacteriosis are totally ignored. This result seems to be linked to a lack of knowledge of food-borne diseases rather than a matter of incidence. Various studies [28,43] have shown that even if pregnant women are generally aware of the existence of the most common infections, a large percentage of women ignore some of them (listeriosis and campylobacteriosis) despite their relevance [1].

In line with previous studies [8,10,44], the coded contents and argumentations showed gaps in knowledge regarding how food-borne diseases are transmitted. For example, toxoplasmosis is confused with listeriosis or salmonellosis with regard to the foods that could transmit it. It is very important to consider these results, as incorrect knowledge can lead to incorrect food behaviour, and is dangerous for mother and foetus.

Most of the exchanges on the social network aim to clarify doubts; expressions of concern and reassurance to such expressions of doubt were highlighted. People in their social environment seek to clarify and resolve ambiguous and often confusing sensations elicited by emotions. Sharing expressions of worry serves to cope with something perceived as uncontrollable by making it meaningful and manageable [27]. The answers to such expressions often deny or minimise the risk, bringing as evidence the respondent experience “I’ve always done it, nothing ever happened”. These types of answers can be attributed to an optimistic bias that frequently occurs in health-related issues [45], influencing food choice and consumption [46]. The belief related to this bias (i.e., to be less exposed to a risk since the absence of issues in the past) may hinder people’s efforts to actuate self-protective behaviours. It is a crucial point that must be considered in risk communication. In fact, people may ignore risk messages “because they believe they are directed to a more vulnerable group (and not to them) and fail to take precautions regarding a hazard” [41] p. 3. Therefore, both perception and correct knowledge on the issue are important, since both can be related to non-protective behaviours [47]. Providing answers that minimise risks as described above, individuals aim to protect themselves from a psychological point of view, rather than inform about the risk in question. By reading a similar experience, people perceived to be exposed to the same risk react in a self-protective way, denying or lessening the danger. An explanation of this behaviour can be found in the protection motivation theory [48] that explains reactions towards a perceived health threat. According to this theory, when fear (because of a threat) is evoked, people feel the urge to reduce that negative emotional state, which can be translated as avoidance or denial [49]. The optimistically biased risk can be the outcome of this reaction that serves as a defensive denial [41].

In other cases, respondents provided an informative answer citing the gynaecologist as the expert. On the one hand, this leads to trust in the professional figures of reference, confirming the literature which maintains that health personnel are considered to be a reliable information source [10] and that pregnant women receive most of the information on food risks from the gynaecologist [28]. However, the gynaecologist is often the only figure mentioned as a reliable source for the existing knowledge on social platforms. This aspect needs to be considered as people need to be aware of the presence of various figures (e.g., infectious disease specialists, microbiologists, dieticians, and nutritionists) and institutions responsible and involved in food safety issues, to have a greater possibility of accessing correct information.

### Limitations

This study has some limitations. The sub-sample qualitatively analysed included few posts. However, this is a result in itself; in fact, the starting corpus was very vast and heterogeneous, and not focused only on food, of which food safety is only a niche. This result provides an idea of how much the topic is addressed in the target audience in reference to the Italian context.

The automatic extrapolation of posts does not allow the maintenance of conversations to observe the interactions between users (they are de-contextualised). Even though the meaning in terms of contents is not lost, it is not possible to change the level of analysis, considering the exchange dynamics. An in-depth study of this aspect (e.g., through a discourse analysis) could be interesting to future researchers. However, without a preliminary automatic analysis, reading the post would be extremely time-consuming, and this step prevented the possibility of keeping the whole conversation. Further studies (not aimed at understanding the extent to which specific topics are dealt with) could specifically collect posts dealing with food behaviours.

Furthermore, we only gathered conversations from public posts/pages, so the analysis is partial, since there may be some closed groups where the topic is debated. Since ethical problems could arise while deciding to conduct a similar study on closed groups in this preliminary study, we only explored the object of interest in public groups/pages. Future research could investigate how to bypass this limitation.

Finally, for this study, we limited our evaluation to the Italian population. Further studies could also contemplate different contexts and cultures.

## 5. Conclusions

This study provided some pivotal insights in the field of food risk communication and food risk knowledge and perception leading to food behaviour. Two main results underlined the need and the importance of tailored communication that clarifies doubts and provides practical indications for food risk management during pregnancy. First, the least known and debated food risks are the most widespread (e.g., campylobacteriosis; cf. [1,50]). This suggests the issues on which communication campaigns directed to food behaviour have to concentrate, and underlies the importance of sensitising the target to such issues. Second, there is a lack of precise knowledge that emerged from the analysed contents. An underestimation or lack of knowledge of food-borne diseases may correspond to an increase in exposure as no measures to contrast them are taken. Moreover, it is necessary to take this result into strong consideration, as wrong or inaccurate information shared and spread on social networks can be amplified and become dangerous. Education and training can assist in this regard, starting from school education.

Three elements, in particular, provided some suggestions on how to sketch out communication campaigns. First, it is important to consider the risk linked to denial and optimistic bias (i.e., the belief that communications are directed to more vulnerable others rather than to themselves; cf. [6,41]), for example explicating the features of the targeted audience. Reducing the perceived social distance between the self and the comparison target can reduce the optimistic bias [51].

Additionally, the importance of relying on experts is reiterated: the gynaecologist is recognised as the main authority regarding this phase of life. However, this result also underlines that all the institutions specifically dedicated to food safety (e.g., zooprophylactic institutes, ASL) need the same visibility to provide people with greater access to information on food risks [30]. The figures who are involved in food risk communication need to build a common strategy so that pregnant women receive clear and useful information on the diet to follow and the best practices to be adopted. The importance of communication also aimed at the continuous training of these figures [52].

The critical role that social networks can assume in seeking and sharing information in front of perceived danger was restated and demonstrated [53]. For this reason, it is crucial that experts and competent authorities exploit this channel to provide accurate information to people.

## Figures and Tables

**Table 1:** Selected groups and pages related to pregnancy.

Thematic Area: Pregnancy	Typology	Number of Like/Members
I consigli delle mamme	page	570,854
Pianeta Mamma	page	363,839
Passione Mamma	page	243,818
Il fantastico mondo delle mamme	page	215,023
Mamme.it	page	157,499
Mamme—mammaoggi	page	138,233
Cose da Mamme—by Silvia Lonardo	page	123,996
Mamme and Dolce Attesa Original Page by Anna	page	109,896
Mamme, pancine and tanto amore	page	104,622
Mamme and Pancione	page	83,857
MammeCreative.it	page	58,046
La rete delle mamme	page	52,570
MammeAcrobate.com	page	26,344
Mamme 40	page	10,458
Mamme gravidanza e alimentazione	page	8046
Ricette per lo svezzamento	group	4472
Pancine Mamme e Bimbi	page	4306
Mamme di Seregno e dintorni	group	2193
Mamme Latina	group	1952
Le Mamme di Mentana	group	1026
Le Mamme Di Padova	group	1010
Mamme Valsesiane e Valsesserine	group	774
MMB—Mamme Monza and Brianza	group	718
Allattamento, Svezzamento e Adolescenza	group	386

**Table 2:** Lexicometric measures of the “Food risks during pregnancy” corpus.

N—Occurrences	24,811
V—Distinct forms	3033
(V/N) × 100—Type/token ratio	12.2
(V1/V) × 100—Hapaxes	57

## Data Availability

The data presented in this study are available on request from the corresponding authors.
